# To what extent does the anxiety scale of the Four-Dimensional Symptom Questionnaire (4DSQ) detect specific types of anxiety disorder in primary care? A psychometric study

**DOI:** 10.1186/1471-244X-14-121

**Published:** 2014-04-24

**Authors:** Berend Terluin, Desiree B Oosterbaan, Evelien PM Brouwers, Annemieke van Straten, Peter M van de Ven, Wendy Langerak, Harm WJ van Marwijk

**Affiliations:** 1 Department of General Practice and Elderly Care Medicine, EMGO Institute for Health and Care Research, VU University Medical Centre, Van der Boechorststraat 7, 1081 BT Amsterdam, The Netherlands; 2 Overwaal Centre for Anxiety Disorders, Radboud University Nijmegen, Nijmegen, The Netherlands; 3 Scientific Centre for Care and Welfare (Tranzo), Tilburg University, Tilburg, The Netherlands; 4 Department of Clinical Psychology, VU University and EMGO Institute for Health and Care Research, VU University Medical Centre, Amsterdam, The Netherlands; 5 Department of Epidemiology and Biostatistics, EMGO Institute for Health and Care Research, VU University Medical Centre, Amsterdam, The Netherlands; 6 Dutch Institute for Employee Benefit Schemes (UWV), Almere, The Netherlands

## Abstract

**Background:**

Anxiety scales may help primary care physicians to detect specific anxiety disorders among the many emotionally distressed patients presenting in primary care. The anxiety scale of the Four-Dimensional Symptom Questionnaire (4DSQ) consists of an admixture of symptoms of specific anxiety disorders. The research questions were: (1) Is the anxiety scale unidimensional or multidimensional? (2) To what extent does the anxiety scale detect specific DSM-IV anxiety disorders? (3) Which cut-off points are suitable to rule out or to rule in (which) anxiety disorders?

**Methods:**

We analyzed 5 primary care datasets with standardized psychiatric diagnoses and 4DSQ scores. Unidimensionality was assessed through confirmatory factor analysis (CFA). We examined mean scores and anxiety score distributions per disorder. Receiver operating characteristic (ROC) analysis was used to determine optimal cut-off points.

**Results:**

Total n was 969. CFA supported unidimensionality. The anxiety scale performed slightly better in detecting patients with panic disorder, agoraphobia, social phobia, obsessive compulsive disorder (OCD) and post traumatic stress disorder (PTSD) than patients with generalized anxiety disorder (GAD) and specific phobia. ROC-analysis suggested that ≥4 was the optimal cut-off point to rule out and ≥10 the cut-off point to rule in anxiety disorders.

**Conclusions:**

The 4DSQ anxiety scale measures a common trait of pathological anxiety that is characteristic of anxiety disorders, in particular panic disorder, agoraphobia, social phobia, OCD and PTSD. The anxiety score detects the latter anxiety disorders to a slightly greater extent than GAD and specific phobia, without being able to distinguish between the different anxiety disorder types. The cut-off points ≥4 and ≥10 can be used to separate distressed patients in three groups with a relatively low, moderate and high probability of having one or more anxiety disorders.

## Background

Several anxiety scales are being employed in research and clinical practice for various reasons. Some scales, often used in research, measure specific types of anxiety (e.g., test anxiety, trait anxiety) or specific aspects of individual anxiety disorders (e.g., worry, social anxiety, specific fears) whereas other scales aim to measure a common characteristic of most, if not all, anxiety states or disorders (i.e., general anxiety) [1]. For use in primary care practice general scales are more relevant because of their promise to detect all or most types of anxiety disorder (i.e., panic disorder, agoraphobia, social phobia, generalized anxiety disorder (GAD), posttraumatic stress disorder (PTSD), obsessive compulsive disorder (OCD) and specific phobia). Detection of anxiety disorders in primary care is important because of their prevalence and associated disability [2]. Research has shown that general practitioners (GPs) recognize a mental health problem in most of their patients with an anxiety disorder but they have difficulty recognizing a specific anxiety disorder [3]. A solution to this problem might be the use of a case finding instrument to distinguish between patients with high risk of having an anxiety disorder and patients with low risk. This tool must be robust to prevalence variations as GPs will use it in patient populations with various prevalence rates.

As relevant studies typically either lump different anxiety disorders together or focus on a limited number of specific anxiety disorders, there is currently a lack of evidence that available and popular anxiety scales are capable of detecting all or most types of anxiety disorder in primary care. Examples of popular anxiety scales are the Hospital Anxiety and Depression Scale (HADS) [4], the Beck Anxiety Inventory (BAI) [5], the anxiety scales of the Depression Anxiety Stress Scale (DASS) [6] and the Mood and Anxiety Symptom Questionnaire (MASQ) [7,8], and the recently developed Generalized Anxiety Disorder scale (GAD-7) [9,10]. The HADS is mainly used in medical settings and appears to perform quite satisfactory [11,12], but it may not detect all relevant types of anxiety disorder (e.g., social phobia) [13-15]. The BAI seems to be biased towards panic disorder [16,17] and failed to detect any anxiety disorder in some studies [18,19]. The anxiety scale of the DASS also seems to favour panic disorder [20]. The anxiety scale of the MASQ was shown to be fairly good in detecting any anxiety disorder in a community sample [21], but in higher prevalence samples the scale discriminated poorly between anxiety disorders and other or no disorders [22,23]. The GAD-7 appears to be a good screener for GAD, panic disorder, social anxiety disorder and PTSD in primary care [9,10], but in higher prevalence samples the GAD-7 performed poorly in detecting GAD [24]. A few studies reported the failure of anxiety scales to discriminate between anxiety and depressive disorders [21,25,26], which may suggest that some anxiety scales actually measure negative affect or general distress [24].

The present study concerns the anxiety scale of the Four-Dimensional Symptom Questionnaire (4DSQ). The 4DSQ is a self-rating questionnaire comprising four scales measuring distress, depression, anxiety and somatization [27]. The anxiety scale is composed of a collection of symptoms that are more or less specific to the various distinct anxiety disorders (see Table 1 for its items). This raises questions about the dimensionality of the anxiety scale. Is the anxiety scale unidimensional, measuring a single trait of anxiety across different groups of patients (e.g., patients with different anxiety and depressive disorders or no disorder), or is the anxiety scale multidimensional, measuring different traits of anxiety in different patient groups (e.g., panic anxiety in panic disorder patients, social anxiety in social phobia patients and general anxiety in GAD patients)? If the 4DSQ anxiety scale is multidimensional, its scores could represent different anxiety problems depending on the specific anxiety disorder involved and anxiety scores could not be compared across diagnostic groups. For instance, an anxiety score of 15 could reflect a totally different problem in a panic disorder patient than in a social phobia patient. From a practical point of view the key question is whether the 4DSQ anxiety scale is able to detect the various specific anxiety disorders equally well (e.g., whether the scale will detect social phobia as well as panic disorder). For the primary care professional it is important to know whether the 4DSQ identifies all anxiety disorders to the same extent or whether it tends to detect some disorders preferentially and miss others.

**Table 1:** **Items of the 4DSQ anxiety scale, mean item scores for the total sample (n = 969)**^**a**^

**Item #**	**Item description**	**Mean item score**
	During the past week, −	
21	- Did you suffer from a vague feeling of fear?	1.04
27	- Did you feel frightened?	0.91
18	- Did you suffer from sudden fright for no reason?	0.73
44	- Were you afraid of becoming embarrassed when with other people?	0.71
24	- Did you suffer from anxiety or panic attacks?	0.64
42	- Were you afraid of anything when there was really no need for you to be afraid? (*for instance animals, heights, small rooms*)	0.52
23	- Did you suffer from trembling when with other people	0.50
50	- Did you have to repeat some actions a number of times before you could do something else?	0.44
40	- Did you have any fear of going out of the house alone?	0.42
45	- Did you ever feel as if you were being threatened by unknown danger?	0.39
49	- Did you have to avoid certain places because they frightened you?	0.36
43	- Were you afraid to travel on buses, streetcars/trams, subways or trains?	0.33

^a^Response options: no (code 0), sometimes (code 1), regularly (code 2), often (code 2), very often or constantly (code 2).

It should be noted that the 4DSQ is not intended to be used as a screening tool in unselected consecutive patients, but rather as an assessment and case finding instrument in emotionally distressed patients. As noted above, GPs usually recognize non-specific emotional problems in patients with an anxiety disorder without recognizing that these patients actually have an anxiety disorder that needs specific treatment [3]. The 4DSQ, as a case finding instrument, could assist GPs in separating patients with high risk of having an anxiety disorder from patients with low risk. The 4DSQ anxiety scale employs two cut-off points, based on clinical experience [28], a lower cut-off point with a relatively high sensitivity and a higher cut-off point with a relatively high specificity. The idea is that the lower cut-off point be used to identify a group of patients (below the cut-off) with a relatively low probability of having an anxiety disorder and that the higher cut-off point be used to identify a group of patients (above the cut-off) with a relatively high probability of having an anxiety disorder. The latter group should be given priority in a subsequent clinical diagnostic workup targeted at anxiety disorder. The current cut-off points (≥8 and ≥13) are probably set too high [29].

The present study evaluated the 4DSQ anxiety scale as a case finding tool to identify anxiety disorder and aimed to answer the following questions: (1) Is the 4DSQ anxiety scale unidimensional or multidimensional and what is the scale’s reliability? (2) To what extent does the 4DSQ anxiety scale detect each of the specific anxiety disorder types? (3) Which cut-off points are suitable to rule out or to rule in (which) anxiety disorders?

## Methods

### Study populations

The design was a cross-sectional secondary analysis of 5 convenience samples collected in different primary care studies (total n = 969). Each of these samples consisted of patients selected for having mental health problems, defined in various ways. Each patient completed the 4DSQ and was subjected to a standardized psychiatric interview administered by trained research assistants. The range of disorders assessed differed across studies.

Dataset A contained the baseline data of general practice patients with emotional distress, who were assessed for eligibility to take part in a randomized clinical trial to investigate the effectiveness of a social work intervention [30]. The diagnostic interview used was the Composite International Diagnostic Interview (CIDI) [31], administered face-to-face. The study was carried out in compliance with the Helsinki Declaration and ethical approval was granted by the Ethical Committee of the Netherlands Institute of Mental Health and Addiction, Utrecht, the Netherlands. Anonymized data were made available by the Netherlands Institute for Health Services Research (NIVEL), Utrecht, the Netherlands.

Dataset B consisted of the baseline data of general practice patients with depressive symptoms, who were assessed for eligibility to participate in a randomized clinical trial to evaluate the effectiveness of antidepressant pharmacotherapy [32]. The CIDI was administered face-to-face. The study was carried out in compliance with the Helsinki Declaration and ethical approval was obtained from the Medical Ethical Committee of the VU University Medical Center, Amsterdam, the Netherlands. Anonymized data were made available by the EMGO Institute for Health and Care Research, Amsterdam, the Netherlands.

Dataset C comprised the baseline data of general practice patients with threshold and subthreshold mood and anxiety disorders, who were included in a randomized clinical trial to assess the effectiveness of a stepped care program [33]. The CIDI was administered by telephone. The study was carried out in compliance with the Helsinki Declaration and ethical approval was obtained from the Medical Ethical Committee of the VU University Medical Center, Amsterdam, the Netherlands (registration number 2006/248). Anonymized data were made available by the Department of Clinical Psychology, VU University, Amsterdam, the Netherlands.

Dataset D consisted of the baseline data of general practice patients who were included in a randomized clinical trial aimed to evaluate a stepped care program for mood, anxiety and stress-related disorders [34]. The diagnostic interview used was the Mini-International Neuropsychiatric Interview (MINI) [35], administered face-to-face. The study was carried out in compliance with the Helsinki Declaration and ethical approval was obtained from the Medical Ethics Committee of the Twenteborg Hospital, Almelo, the Netherlands. Anonymized data were made available by Desiree B. Oosterbaan.

Dataset E was derived from a cross-sectional survey among employees who had been unable to work for more than two years due to mental health problems and who applied for a work disability benefit according to Dutch regulations [36]. The diagnostic interview consisted of the CIDI, administered face-to-face. The study was carried out in compliance with the Helsinki Declaration and ethical approval was obtained from the Medical Ethical Committee of the VU University Medical Center, Amsterdam, the Netherlands. Anonymized data were made available by the Department of Psychiatry, VU University Medical Center, Amsterdam, the Netherlands.

It should be noted that the selected patient samples were all more or less representative of the so called “indicated” population [37], the population in which the 4DSQ anxiety scale is indicated to contribute to the separation of patients with and without anxiety disorder.

### Measures

#### Four-Dimensional Symptom Questionnaire (4DSQ)

The 4DSQ has been developed in primary care as a tool to detect mental health problems, assess overall severity, and select patients with a high risk of having a depressive or anxiety disorder. Importantly, the 4DSQ dimensions were empirically derived through factor and cluster analysis of a pool of 96 symptoms covering the whole range of non-psychotic psychological and psychosomatic symptoms, without prior assumptions about the number and nature of the dimensions [38]. The 4DSQ comprises four scales measuring distress, depression, anxiety and somatization [27]. It takes on average 5–10 minutes to complete. The anxiety scale consists of 12 items measuring irrational fears, panic, avoidance, and other features associated with anxiety disorders (see Table 1). The scale’s reliability is generally good with Cronbach’s alpha values generally well over 0.80. Response categories are “no”, “sometimes”, “regularly”, “often”, “very often or constantly”, which are scored as 0 for “no”, 1 for “sometimes” and 2 for the other response categories. Item scores are summated to obtain scale scores. The rationale behind collapsing the highest response categories “regularly”, “often”, “very often or constantly” into a single score category is to avoid spurious correlations due to exaggerating response tendencies. This way of scoring ensures that the scale score reflects primarily the number of symptoms rather than their subjective severity [39]. The 4DSQ is freely available for non-commercial use as in health care and research [40].

#### Standardized psychiatric interview

The studies employed two different diagnostic interviews, the Composite International Diagnostic Interview (CIDI) and the Mini-International Neuropsychiatric Interview (MINI). The CIDI is a structured interview suitable to be applied by trained lay interviewers [31]. It allows standardized diagnoses of mental disorders according to the definitions of the ICD-10 and DSM-IV (we used DSM-IV diagnoses only). Reliability and validity are generally good [41]. The MINI is also a structured interview, but is it shorter than the CIDI [35]. The MINI has good reliability and agreement with the CIDI [42]. As both interviews are known to produce reliable and valid DSM-IV diagnoses, we assumed that the CIDI and the MINI interviews produced equivalent results. That is, we assumed that, for instance, panic disorder diagnosed in one study using the CIDI was essentially the same disorder as panic disorder diagnosed in another study using the MINI, although differences in prevalence and severity across the studies might have existed. There was no way to test our assumption of invariant diagnoses across studies, we simply had to rely on it. However, it should be noted that major violation of this assumption (e.g., when panic disorder according to the CIDI was a different condition than panic disorder according to the MINI) would have resulted in significantly decreased psychometric parameter estimates after pooling the samples as the 4DSQ anxiety score would have been compared to a hodgepodge of different conditions.

### Analysis

To describe the study samples, we examined the composition of the samples regarding the prevalence of specific disorders, the occurrence of multiple anxiety disorders and comorbidity between anxiety and depressive disorders.

All analyses were performed in the five study samples separately and, where possible, in the pooled sample of five studies (total n = 969). Some anxiety disorders were only assessed in two studies; in these cases the pooled analyses were limited to the studies in which the specific anxiety disorder was assessed.

To assess the dimensionality of the 4DSQ anxiety scale we examined the fit indices of a one factor model using confirmatory factor analysis (CFA) in the five studies separately. Fit indices examined were the χ^2^/df statistic, the Root Mean Square Error of Approximation (RMSEA), the Comparative Fit Index (CFI) and the Tucker-Lewis Index (TLI). RMSEA values less than 0.08, χ^2^/df statistics less than 3, and CFI and TLI values greater than 0.95 were accepted as indicating adequate fit [43]. Strict factorial invariance across all five studies was tested using a multi-group CFA. The fit of the strict factorial invariance was compared to a partial factorial invariance model (in which the residual variances were allowed to differ between studies) using the χ^2^ test. CFA and multi-group CFA analyses were performed in M-plus version 7 using theta parameterisation [43].

As a measure of internal consistency reliability we determined the anxiety scale’s Cronbach’s alpha. We calculated the anxiety scale’s standard error of measurement (SEM) from the scale’s standard deviation (SD) and the alpha coefficient, using the formula

$$\mathrm{SEM}=\mathrm{SD}\;x{\left(1–\mathrm{alpha}\right)}^{1/2}.$$

The SEM, being the standard deviation of the measurement error of the scale score, allows an estimation of the confidence interval around individual scores. This information is useful for choosing and interpreting practical cut-off points for the scale.

To assess the extent to which the 4DSQ anxiety scale was able to detect the various specific anxiety disorders, we explored the anxiety score distributions by drawing boxplots for the individual anxiety disorders, for patients with single and multiple anxiety disorders, and for patients with depressive disorder(s) only, anxiety disorder(s) only, and comorbid anxiety-depressive disorders. In addition, we calculated mean anxiety scores and standard deviations for the various diagnostic groups. Differences between groups were tested using the non-parametric Kruskal-Wallis test to account for the skewed score distribution in some of the groups. Pair-wise post hoc tests were performed using the software package “pgirmess” as implemented in the statistical program R version 3.0.1 [44].

To determine optimal cut-off points for the 4DSQ anxiety scale we performed receiver operating characteristic (ROC) analyses with the anxiety score as the test variable and anxiety disorders as the state variable, in the separate studies and in the pooled samples. As it turned out that the anxiety score seemed to be more consistently associated with panic disorder, agoraphobia, social phobia, OCD and PTSD than with GAD and specific phobia, we performed ROC analyses with the former five disorders as state variable. Because only panic disorder, agoraphobia and social phobia had been assessed in all five studies, we first performed a ROC analysis with these three disorders as outcome variable. We determined the best ROC thresholds, being the thresholds closest to the top-left corner of ROC graph (i.e., sensitivity = 1, 1–specificity = 0). In addition, we determined the highest thresholds with an arbitrarily chosen sensitivity of ≥0.85, possibly suitable as the lower cut-off point of the scale to rule out anxiety disorder when the test is negative, and the thresholds with an arbitrarily chosen specificity of ≥0.85, possibly suitable as the higher cut-off point of the scale to rule in anxiety disorder when the test is positive. We used package “pROC” as implemented in R to perform the ROC analyses and to estimate 95% confidence intervals (95% CI) of the thresholds and operational parameters using bootstrapping (2000 samples) [45]. Next, the analysis was repeated with panic disorder, agoraphobia, social phobia, OCD or PTSD as outcome variable in the samples in which the latter two disorders had been assessed.

A set of thresholds was chosen using all available information. Finally, we calculated likelihood ratios to evaluate the performance of these thresholds with respect to the detection of panic disorder, agoraphobia and social phobia, as well as to the detection of any anxiety disorder. The likelihood ratio (LR) of a test result (e.g., a certain anxiety score or a range of scores) is the ratio between the probability of this result in a population with the diagnosis of interest (e.g., anxiety disorder) and the probability of this result in a population without the diagnosis of interest [46]. LRs are relatively independent of the prevalence of the diagnosis of interest in the study population. Once LRs are known, the probability of a diagnosis, given a certain test result and a certain prevalence, can be calculated relatively easy because the LR is also the ratio between the posterior odds of having a disorder and the prior odds of having the disorder, with the latter simply being the prevalence divided by 1 minus the prevalence. The posterior probability of having a disorder is the posterior odds divided by 1 plus the posterior odds [46]. We have calculated LRs for the defined low, moderate and high anxiety scores based on the pooled sample. Subsequently, we used these LRs to calculate the predictive value of low, moderate and high anxiety scores with respect to ruling in or ruling out panic disorder, agoraphobia and social phobia, and any anxiety disorder respectively, in two hypothetical samples, one similar to our pooled sample, the other with half the prevalence of anxiety disorder. LRs and their confidence intervals were calculated using the website for statistical computation VassarStats (http://vassarstats.net/).

The analyses, other than the CFAs, the ROC-analyses and the post hoc analyses after the Kruskal-Wallis tests, were performed using SPSS 20.0.

## Results

### Prevalence and comorbidity

Details of the study samples are presented in Table 2. The diagnostic composition of the samples varied to some extent. Studies that focused on the whole spectrum of depressive and anxiety disorders (studies C, D and E) selected relatively more patients with anxiety disorders. Study B that focused on patients with depressive complaints included relatively more patients with depressive disorders and fewer patients with anxiety disorder, except for GAD. We refrained from formal statistical testing of these between-study differences because generalization of these differences would serve no purpose. It suffices to note that there was some heterogeneity between the study samples, which likely resulted from the different settings and purposes for which the samples had been collected.

**Table 2:** Description of the datasets

**Study**	**A**	**B**	**C**	**D**	**E**
Numbers^a^	295	170	118	156	230
Gender (% female)	60.3	72.4	64.4	61.5	67.8
Age [mean (SD)]	39.5 (9.2)	44.9 (15.9)	49.5 (11.2)	38.1 (12.1)	43.5 (7.5)
4DSQ scores [mean (SD)]					
- Distress (range 0–32)	22.9 (7.2)	21.0 (7.5)	19.4 (7.1)	19.8 (9.2)	20.7 (9.0)
- Depression (range 0–12)	4.0 (3.4)	4.3 (3.8)	3.7 (3.1)	4.4 (4.0)	4.9 (4.4)
- Anxiety (range 0–24)	5.9 (5.4)	5.9 (5.8)	7.0 (5.2)	6.6 (6.3)	9.4 (7.3)
- Somatization (0–32)	14.2 (6.9)	12.7 (7.2)	14.2 (6.9)	12.0 (8.0)	14.7 (7.7)
DSM-IV diagnoses (%)					
- Panic disorder^b^	7.8	8.8	26.3	26.3	28.7
- Agoraphobia^c^	7.1	12.4	32.2	21.8	27.0
- Social phobia	10.8	7.1	22.9	21.2	23.5
- GAD^d^	20.7	32.9	18.6	16.7	26.1
- Specific phobia	N/A	17.1	N/A	8.3	N/A
- OCD^e^	N/A	N/A	N/A	5.1	17.0
- PTSD^f^	N/A	N/A	N/A	3.8	20.0
- Major depressive disorder	49.5	67.1	42.4	50.6	40.0
- Dysthymia	2.4	18.2	12.7	3.8	28.7

^a^Numbers of patients refer to those who completed the 4DSQ and a diagnostic interview.

^b^Panic disorder without agoraphobia plus panic disorder with agoraphobia.

^c^Agoraphobia without history of panic disorder plus panic disorder with agoraphobia.

^d^Generalized anxiety disorder.

^e^Obsessive compulsive disorder.

^f^Post traumatic stress disorder.

N/A: not assessed.

Table 3 shows the prevalence of multiple anxiety disorders and the co-occurrence of anxiety and depressive disorders (anxiety-depression comorbidity). For instance, of all patients across the study samples diagnosed with panic disorder (n = 176) 86% had one or more other anxiety disorders too, and 59% of the panic disorder patients had a comorbid depressive disorder (i.e., major depressive disorder or dysthymia). For each of the specific anxiety disorders, the occurrence of multiple anxiety disorders (56-88%) and anxiety-depression comorbidity (55-74%) was more the rule than an exception. Of all patients with one or more anxiety disorders (n = 477), 228 (48%) had a single anxiety disorder, which was most frequently (in 99 cases) GAD. It should be noted that the already high prevalence of multiple anxiety disorders was probably underestimated to some extent because specific phobia, OCD and PTSD had not been assessed in three studies (see Table 2).

**Table 3:** Occurrence of multiple anxiety disorders and anxiety-depression comorbidity, over the study samples pooled and the range across the separate study samples

**Disorder**	**N**	**Multiple anxiety disorders (%)**^**a**^		**Anxiety-depression comorbidity (%)**^**a**^	
		**pooled**	**range**	**pooled**	**range**
Panic disorder^b^	176	86	70-96	59	39-80
Agoraphobia^c^	176	88	71-95	56	37-76
Social phobia	158	71	58-80	63	41-83
GAD^d^	225	56	30-80	66	50-79
Specific phobia	42	59	46-66	74	46-86
OCD^e^	47	85	82-100	55	54-63
PTSD^f^	52	73	72-83	63	61-83

^a^Percentages of patients with specific anxiety disorders who also had one or more other anxiety disorders or comorbid depressive disorder.

^b^Panic disorder without agoraphobia plus panic disorder with agoraphobia.

^c^Agoraphobia without history of panic disorder plus panic disorder with agoraphobia.

^d^Generalized anxiety disorder.

^e^Obsessive compulsive disorder.

^f^Post traumatic stress disorder.

### Unidimensionality and reliability

The results of the CFAs are displayed in Table 4. For all studies separately the one factor model showed adequate fit. Moreover, for the studies combined, the strict factorial invariance model showed adequate fit on all indices. Fit of the strict factorial invariance model was not worse than that of the partial factorial invariance model (χ^2^ = 57.6, df = 48, p = 0.162).

**Table 4:** Fit indices of single factor model for studies separately and for the strict and partial factorial invariance model for all studies together

**Study**	**χ**^**2**^	**df**	**p**	**χ**^**2**^**/df**	**RMSEA**	**CFI**	**TLI**
Study A	156.6	54	<0.0001	2.9	0.080	0.966	0.958
Study B	115.6	54	<0.0001	2.1	0.082	0.966	0.958
Study C	83.0	54	0.0068	1.5	0.067	0.971	0.964
Study D	88.4	54	0.0022	1.6	0.064	0.986	0.983
Study E	87.6	54	0.0026	1.6	0.052	0.993	0.991
Studies A-E strict factorial invariance model	639.3	406	<0.0001	1.6	0.054	0.983	0.986
Studies A-E partial factorial invariance model	645.7	358	<0.0001	1.8	0.064	0.979	0.981

Cronbach’s alpha varied between 0.85 (study C) and 0.92 (study E) and was 0.90 in the pooled sample. The anxiety scale’s standard deviation varied between 5.2 (study C) and 7.3 (study E) and was 6.2 in the pooled sample. The SEM varied between 1.9 (studies A and B) and 2.0 (studies C-E) and was 2.0 in the pooled sample. This value of SEM means that, due to measurement error, the 96% confidence interval of a given score X was X ± 4 points and that the 84% confidence interval of a given score X was X ± 3 points.

### Anxiety score distributions

**Figure 1:**
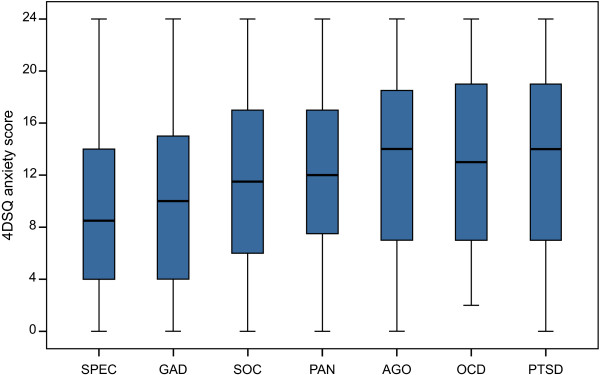
**Disorder-specific 4DSQ anxiety score distributions.** Separate boxplots for specific phobia (SPEC), generalized anxiety disorder (GAD), social phobia (SOC), panic disorder (PAN), agoraphobia (AGO), obsessive-compulsive disorder (OCD) and posttraumatic stress disorder (PTSD). The boxes of the boxplots represent the median scores and the interquartile ranges. The whiskers of the plots reach to the extreme scores.

The boxplots depicting the disorder-specific anxiety score distributions (Figure 1) suggest a difference in overall level of anxiety, as measured by the 4DSQ anxiety scale, between GAD and specific phobia on the one hand and panic disorder, agoraphobia, social phobia, OCD and PTSD on the other hand. It appeared that, on average, panic disorder, agoraphobia, social phobia, OCD and PTSD were characterized by slightly higher anxiety scores than GAD and specific phobia.

**Figure 2:**
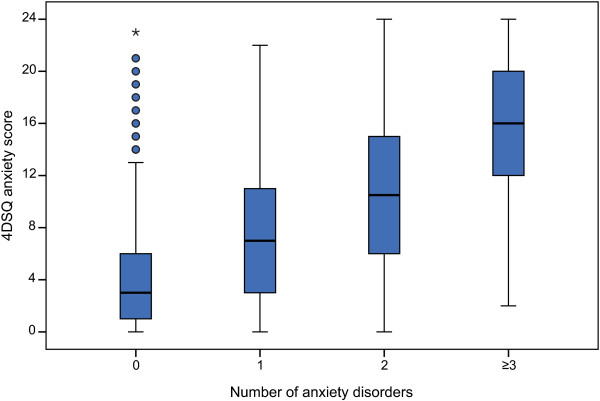
**4DSQ anxiety score distributions by number of anxiety disorders per patient.** The boxes of the boxplots represent the median scores and the interquartile ranges (IQR). The whiskers of the plots reach to the extreme scores, with a maximum distance to the box of 1.5 IQR. Outliers are represented by dots (distance to the box of 1.5-3 IQR) or asterisks (distance to the box of >3 IQR).

Figure 2 demonstrates the differences in anxiety score associated with the number of anxiety disorders per patient. The median anxiety score for patients with single anxiety disorders was 7 whereas the median score for patients with three or more anxiety disorders was 16. Clearly, the anxiety score was an indicator of the number of anxiety disorders per patient. Of the patients with three or more anxiety disorders over 50% scored very high (i.e., ≥16) and less than 10% scored low (i.e., <4). In contrast, no more than 10% of patients with single anxiety disorders scored very high (i.e., ≥16) and 29% scored low (i.e., <4). As noted above, GAD was the most frequent diagnosis in the single anxiety disorder group (43%). Also relevant for the ability of the anxiety score to detect anxiety disorders was the finding that only 11% of patients without a diagnosed anxiety disorder scored ≥10. An anxiety score ≥10 indicated a relatively high probability of having one or more anxiety disorders. Note that Figure 2 does not account for comorbidity with depressive disorder.

**Figure 3:**
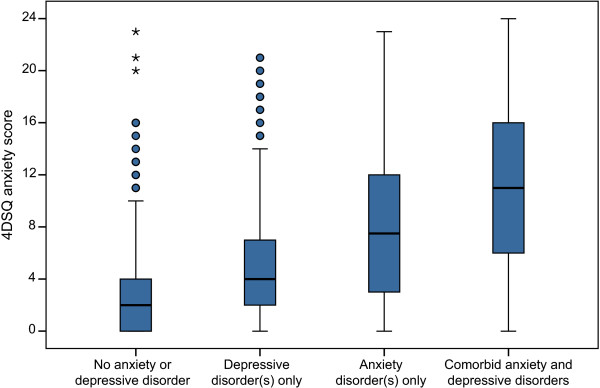
**4DSQ anxiety score distributions by comorbidity groups.** The boxes of the boxplots represent the median scores and the interquartile ranges (IQR). The whiskers of the plots reach to the extreme scores, with a maximum distance to the box of 1.5 IQR. Outliers are represented by dots (distance to the box of 1.5-3 IQR) or asterisks (distance to the box of >3 IQR).

Anxiety-depression comorbidity was also strongly related with the anxiety score distribution (Figure 3). Of the patients with non-comorbid anxiety disorders 27% scored low (i.e., <4) on the anxiety scale and 54% of them had a single anxiety disorder. The presence of depressive disorder was also associated with an increase in the anxiety score, although a smaller increase than the increase associated with the presence of anxiety disorder.

Mean anxiety scores per disorder are displayed in Table 5. The highest mean scores occurred in patients with panic disorder, agoraphobia, OCD, PTSD and social phobia. The mean anxiety score appeared to be strongly associated with the number of anxiety disorders per patient (Kruskal-Wallis test p <0.001). To account for multiple pair-wise comparisons between four groups (6 comparisons) we adopted a critical p-value of 0.0083 (0.05/6) for the post hoc tests. All between group tests were significant (p <0.0083). By the same token, the comorbidity groups were significantly different (Kruskal-Wallis test p < 0.001). Post hoc tests (with the same adjustment for multiple testing) revealed that all between-group differences were significant (p <0.0083).

**Table 5:** Mean anxiety scores per anxiety disorder, per number of anxiety disorders and per comorbidity group, over the study samples pooled and the range across the separate study samples

	**Pooled**			**Range**	
	**N**	**Mean**	**(SD)**	**Mean**	**(SD)**
Anxiety disorder					
- Panic disorder^a^	176	12.6	(6.2)	9.8 – 14.8	(4.2 – 7.5)
- Agoraphobia^b^	176	12.9	(6.6)	9.0 – 15.5	(5.4 – 6.6)
- Social phobia	158	11.9	(6.8)	9.3 – 15.1	(5.4 – 7.4)
- GAD^c^	225	9.9	(6.5)	8.0 – 13.7	(5.1 – 6.9)
- Specific phobia	42	9.5	(6.4)	7.6 – 10.4	(6.4 – 6.4)
- OCD^d^	47	12.8	(7.0)	7.8 – 13.9	(4.4 – 7.0)
- PTSD^e^	52	12.9	(6.7)	10.7 – 13.2	(5.0 – 6.9)
Number of anxiety disorders					
- 0	492	4,1	(4,4)	3.1 – 4.7	(3.9 – 5.3)
- 1	228	7,4	(5,3)	6.0 – 8.5	(4.3 – 6.3)
- 2	148	10,6	(5,8)	8.6 – 12.3	(5.0 – 6.3)
- ≥ 3	101	15,1	(6,0)	12.4 – 17.3	(5.0 – 6.5)
Comorbidity groups					
- No anxiety or depressive disorder	263	3.2	(4.0)	1.8 – 5.4	(2.7 – 4.9)
- Depressive disorder(s)^f^ only	229	5.0	(4.7)	4.1 – 9.2	(3.7 – 5.9)
- Anxiety disorder(s) only	198	8.3	(5.9)	6.5 – 9.9	(5.2 – 6.5)
- Comorbid anxiety and depressive disorders	279	11.3	(6.5)	8.6 – 14.2	(4.7 – 6.7)

^a^Panic disorder without agoraphobia plus panic disorder with agoraphobia.

^b^Agoraphobia without history of panic disorder plus panic disorder with agoraphobia.

^c^Generalized anxiety disorder.

^d^Obsessive compulsive disorder.

^e^Post traumatic stress disorder.

^f^Major depressive disorder and/or dysthymia.

In conclusion, the anxiety scale appeared to detect multiple anxiety disorders better than single anxiety disorders, comorbid anxiety-depressive disorders better than non-comorbid anxiety disorders, and panic disorder, agoraphobia, social phobia, OCD and PTSD better than GAD and specific phobia.

### ROC analysis

ROC analysis with panic disorder, agoraphobia and social phobia as the outcome variable revealed area under the curve (AUC) values in the separate studies between 0.737 and 0.857 (Table 6). In the pooled sample the AUC was 0.793 (95% CI 0.763 – 0.822) indicating that the overall diagnostic accuracy was fair [47]. The best ROC threshold nearest to the top-left corner of the ROC-graph (i.e., sensitivity =1 and 1–specificity = 0) varied between 4.5 and 8.5. Note that ROC-analysis defines thresholds in between observed scores, hence a threshold of 4.5 is equivalent to a cut-off point ≥5. The best ROC threshold in the pooled sample was 6.5 (95% CI 5.5–7.5). The lower threshold with sensitivity ≥0.85 varied between 1.5 and 5.5 across the studies, and was 3.5 in the pooled sample. The higher threshold with specificity ≥0.85 varied between 8.5 and 15.5, and was 10.5 in the pooled sample. The thresholds demonstrated substantial variability across the studies. Note also that the confidence intervals of the thresholds in the individual studies were rather wide, depending on the sample size. In the pooled sample the confidence interval of the threshold was reduced to ±1. Note further that the lower threshold was associated with a high negative predictive value (npv) of 0.89 (0.86–0.92) in the pooled sample, and that the higher threshold was associated with a relatively high positive predictive value (ppv) of 0.65 (0.59–0.70) for the detection of panic disorder, agoraphobia or social phobia. The repeated analysis with panic disorder, agoraphobia, social phobia, OCD or PTSD as the outcome variable showed an AUC in the pooled sample of studies D and E of 0.807 (95% CI 0.761–0.848). The best ROC threshold was 6.5 (4.5–8.5) with sensitivity of 0.73 (0.67–0.78), specificity of 0.75 (0.69–0.81), ppv of 0.77 (0.73–0.82) and npv of 0.70 (0.65–0.75). The lower threshold was 2.5 (1.5–3.5) with sensitivity of 0.88 (0.84–0.93), specificity of 0.47 (0.39–0.55), ppv of 0.66 (0.63–0.70) and npv of 0.78 (0.71–0.85). The higher threshold was 9.5 (7.5–11.5) with sensitivity of 0.60 (0.53–0.66), specificity of 0.86 (0.81–0.91), ppv of 0.83 (0.78–0.89) and npv of 0.65 (0.61–0.69). Note the increase of the ppv (from 0.65 to 0.83) of the higher threshold by the inclusion of OCD and PTSD in the outcome, despite the use of a 1 point lower threshold.

**Table 6:** Results of the ROC-analysis with panic disorder/agoraphobia/social phobia as outcome variable

**Study**	**A**	**B**	**C**	**D**	**E**	**Pooled A-E**
Total numbers	295	170	118	156	230	969
Prevalence^a^	19.0%	16.5%	50.0%	48.7%	42.6%	32.7%
AUC^b^ (95% CI)	.851 (.803; .896)	.857 (.774; .927)	.737 (.643; .820)	.787 (.714; .855)	.775 (.714; .834)	.793 (.763; .822)
Best threshold^c^ (95% CI)	7.5 (5.5; 8.5)	8.5 (4.5; 12.5)	5.5 (4.5; 8.5)	4.5 (3.5; 6.5)	7.5 (6.5; 11.5)	6.5 (5.5; 7.5)
- Sensitivity (95% CI)	.79 (.68; .89)	.71 (.54; .86)	.71 (.59; .83)	.75 (.64; .84)	.79 (.70; .86)	.74 (.69; .79)
- Specificity (95% CI)	.77 (.72; .83)	.84 (.77; .89)	.63 (.51; .75)	.73 (.63; .83)	.67 (.59; .75)	.71 (.67; .75)
- ppv^d^ (95% CI)	.45 (.39; .52)	.47 (.36; .59)	.63 (.58; .75)	.72 (.65; .80)	.64 (.58; .70)	.55 (.52; .59)
- npv^e^ (95% CI)	.94 (.91; .97)	.94 (.90; .97)	.69 (.60; .78)	.75 (.68; .83)	.81 (.75; .87)	.85 (.82; .87)
Lower threshold^f^ (95% CI)	5.5 (3.5; 7.5)	4.5 (1.5; 8.5)	2.5 (1.5; 4.5)	1.5 (0.5; 3.5)	3.5 (2.5; 7.5)	3.5 (2.5; 4.5)
- Sensitivity (95% CI)	.86 (.75; .95)	.89 (.79; 1.00)	.88 (.80; .97)	.91 (.84; .96)	.87 (.80; .93)	.87 (.83; .90)
- Specificity (95% CI)	.68 (.62; .74)	.62 (.54; .70)	.37 (.25; .49)	.43 (.31; .54)	.42 (.33; .50)	.51 (.47; .55)
- ppv^d^ (95% CI)	.39 (.34; .44)	.32 (.27; .37)	.58 (.53; .64)	.60 (.55; .65)	.52 (.49; .57)	.46 (.44; .49)
- npv^e^ (95% CI)	.95 (.92; .98)	.97 (.93; 1.00)	.76 (.61; .91)	.83 (.72; .93)	.81 (.73; .89)	.89 (.86; .92)
Higher threshold^g^ (95% CI)	10.5 (8.5; 11.5)	9.5 (7.5; 11.5)	9.5 (7.5; 12.5)	8.5 (6.5; 12.5)	15.5 (11.5; 17.5)	10.5 (9.5; 11.5)
- Sensitivity (95% CI)	.57 (.45; .70)	.64 (.46; .82)	.42 (.31; .56)	.50 (.38; .61)	.43 (.32; .52)	.52 (.46; .57)
- Specificity (95% CI)	.89 (.85; .93)	.86 (.80; .92)	.86 (.78; .95)	.89 (.81; .95)	.87 (.81; .92)	.86 (.83; .89)
- ppv^d^ (95% CI)	.54 (.45; .65)	.47 (.36; .61)	.76 (.63; .89)	.81 (.71; .91)	.71 (.61; .82)	.65 (.59; .70)
- npv^e^ (95% CI)	.90 (.87; .93)	.92 (.89; .96)	.60 (.54; .66)	.65 (.60; .71)	.67 (.63; .71)	.79 (.77; .81)

^a^Prevalence of panic disorder/agoraphobia/social phobia.

^b^Area under the ROC curve.

^c^Threshold closest to top-left corner of ROC graph (i.e., sensitivity = 1, (1-specificity) = 0).

^d^Positive predictive value.

^e^Negative predictive value.

^f^Lower threshold = highest threshold with sensitivity ≥0.85.

^g^Higher threshold = lowest threshold with specificity ≥0.85.

### Likelihood ratios and predicted probabilities

Based on the ROC-analyses, we decided to choose ≥4 and ≥10 as the revised lower and higher cut-off points for the 4DSQ anxiety scale. The lower cut-off point (≥4) identified 85-90% of all patients with panic disorder, agoraphobia, social phobia, OCD or PTSD and 80% of all patients with GAD or specific phobia. The higher cut-off point (≥10) identified two thirds of all patients with panic disorder, agoraphobia, social phobia, OCD or PTSD and 50% of all patients with GAD or specific phobia. Anxiety disorder patients who scored low (<4) on the anxiety scale, consisted mainly of patients with single anxiety disorders (73%) and patients with non-comorbid disorders (60%) whereas 50% of them had GAD. The percentage of patients with anxiety scores 4–9 varied between 25% and 42% in the separate study samples, and was 31% in the pooled sample.

The LRs associated with low, moderate and high anxiety scores are presented in Table 7. The prevalence of panic disorder, agoraphobia and social phobia in the pooled sample was 317/969 = 32.7%. The likelihood of scoring 0–3 among patients with panic disorder, agoraphobia and social phobia was 42/317 = 0.132, whereas this likelihood among patients without panic disorder, agoraphobia and social phobia was 332/652 = 0.509. The ratio of these likelihoods was 0.132/0.509 = 0.259. Table 8 presents the predicted probabilities of having panic disorder, agoraphobia or social phobia, or any anxiety disorder respectively, based on the LR and the 4DSQ anxiety score. The probabilities were calculated using the following equations:

$$\mathrm{prior}\;\mathrm{odds}=\mathrm{prevalence}/\left(1–\mathrm{prevalence}\right)$$

posteriorodds=priorodds×LR

$$\mathrm{probability}=\mathrm{posterior}\;\mathrm{odds}/\left(1+\mathrm{posterior}\;\mathrm{odds}\right).$$

**Table 7:** Likelihoods and likelihood ratios associated with low (0–3), moderate (4–9) and high (10–24) 4DSQ anxiety scores with respect to panic disorder, agoraphobia and social phobia, and to any anxiety disorder respectively

		**Panic disorder, agoraphobia and social phobia**					**Any anxiety disorder**				
**Anxiety score**	**Total N**	**Disorder**		**No disorder**			**Disorder**		**No disorder**		
		**N**	**LH**^**a**^	**N**	**LH**^**a**^	**LR (95% CI)**^**b**^	**N**	**LH**^**a**^	**N**	**LH**^**a**^	**LR (95% CI)**^**b**^
0-3	374	42	0.13	332	0.51	0.26 (0.19; 0.35)	90	0.19	284	0.58	0.33 (0.27; 0.40)
4-9	301	92	0.29	209	0.32	0.91 (0.74; 1.11)	145	0.30	156	0.32	0.96 (0.79; 1.16)
10-24	294	183	0.58	111	0.17	3.39 (2.79; 4.12)	242	0.51	52	0.11	4.80 (3.66; 6.30)
Total	969	317	1	652	1		477	1	492	1	

^a^LH = likelihood of anxiety score within the Anxiety disorder group and No anxiety disorder group respectively.

^b^LR = likelihood ratio.

**Table 8:** Predicted probabilities for panic disorder, agoraphobia and social phobia, and for any anxiety disorder respectively based on the prevalence and likelihood ratios associated with low (0–3), moderate (4–9) and high (10–24) 4DSQ anxiety scores

	**Panic disorder, agoraphobia and social phobia**			**Any anxiety disorder**		
**Anxiety score**	**LR (95% CI)**^**c**^	**Prevalence = 0.33**^**a **^**(prior odds = 0.49)**	**Prevalence = 0.16**^**b **^**(prior odds = 0.19)**	**LR (95% CI)**^**c**^	**Prevalence = 0.49**^**a **^**(prior odds = 0.96)**	**Prevalence = 0.25**^**b **^**(prior odds = 0.33)**
		**Prob. (95% CI)**^**d**^	**Prob. (95% CI)**^**d**^		**Prob. (95% CI)**^**d**^	**Prob. (95% CI)**^**d**^
0-3	0.26 (0.19; 0.35)	0.12 (0.08; 0.15)	0.05 (0.04; 0.07)	0.33 (0.27; 0.40)	0.24 (0.21; 0.28)	0.10 (0.08; 0.12)
4-9	0.91 (0.74; 1.11)	0.31 (0.26; 0.35)	0.15 (0.12; 0.17)	0.96 (0.79; 1.16)	0.48 (0.43; 0.53)	0.24 (0.21; 0.28)
10-24	3.39 (2.79; 4.12)	0.62 (0.58; 0.67)	0.39 (0.35; 0.44)	4.80 (3.66; 6.30)	0.82 (0.78; 0.86)	0.61 (0.55; 0.68)

^a^Prevalence based on the pooled sample in this study.

^b^Half the prevalence in the pooled sample in this study.

^c^LR = likelihood ratio.

^d^Prob. = predicted posterior probability of disorder given the prevalence and anxiety score.

As expected, low anxiety scores (0–3) predicted relatively low probabilities of having an anxiety disorder and high anxiety scores (10–24) predicted relatively high probabilities, depending on the prevalence of anxiety disorder. Note that low anxiety scores are relatively good in ruling out panic disorder, agoraphobia and social phobia but perform relatively poorly in ruling out any anxiety disorder, especially in high prevalence samples. The obvious reason is that, as we have seen before, about one fifth of patients with GAD or specific phobia have low anxiety scores. On the other hand, high anxiety scores do a relatively good job in ruling in (any) anxiety disorder. The LRs associated with moderate anxiety scores (4–9) were close to 1 and, consequently, the posterior probability was close to the prevalence of anxiety disorder. Moderate anxiety scores are little informative.

## Discussion

Our results suggest that, in primary care patients, the 4DSQ anxiety scale measures a unidimensional construct. In other words, the scale seems to measure a common trait of anxiety symptoms that is present to a lesser or greater extent in various patient groups. This common trait of pathological anxiety appears to be present to a greater extent in patients with panic disorder, agoraphobia, social phobia, OCD and PTSD, and to a slightly lesser extent in patients with GAD and specific phobia. It is absent, or present to a relatively small extent, in patients with non-comorbid depressive disorders and in emotionally distressed patients without any anxiety or depressive disorder. Notwithstanding the fact that the 4DSQ anxiety scale consists of an admixture of vague anxiety symptoms (e.g., vague feeling of fear, feeling frightened) and symptoms that are more or less specific to distinct anxiety disorder types (e.g., anxiety or panic attacks, irrational specific fears, fear of public embarrassment, repeating actions, avoiding places, fear of public transport) the anxiety scale symptoms appear to work together to measure a common dimension of pathological anxiety. Although the specific anxiety disorders are conceptualized as separate disorders in DSM-IV, in our samples, the specific anxiety disorders relatively rarely occurred stand-alone as single disorders. Multiple anxiety disorders were the rule, rather than an exception. This might, in part, explain why we found the anxiety scale to be unidimensional. Additional research is needed to clarify the dimensions of anxiety symptoms and disorders.

The kind of anxiety that is measured by the 4DSQ anxiety scale was present in most patients with anxiety disorders. This finding compares favourably to existing anxiety scales. However, this anxiety was present to a slightly greater extent in patients with panic disorder, agoraphobia, social phobia, OCD or PTSD than in patients with GAD or specific phobia, and undeniably it was present to a greater extent in patients with multiple anxiety disorders than in patients with single anxiety disorders, and in comorbid anxiety-depressive disorders than in non-comorbid anxiety disorders. Still, the majority of patients with GAD or specific phobia (79%), single anxiety disorders (71%) and non-comorbid anxiety disorders (73%) scored at or above the lowest cut-off point (≥4). Nevertheless, 20-30% of these disorders scored low (<4). In contrast, 85-90% of patients with panic disorder, agoraphobia, social phobia, OCD or PTSD, multiple anxiety disorders or comorbid anxiety-depressive disorders scored ≥4. When it comes to detecting anxiety disorders in primary care patients, the 4DSQ performs better with respect to panic disorder, agoraphobia, social phobia, OCD or PTSD, multiple anxiety disorders and comorbid anxiety-depressive disorders.

A sufficiently strong association between the 4DSQ anxiety score and the presence of anxiety disorder constitutes a prerequisite for the anxiety score to be useful as a tool to detect anxiety disorder. This association depends, first of all, on the concordance of whatever the anxiety scale is measuring and what characterizes anxiety disorder (a matter of validity). In the hypothetical situation that there is 100% concordance, all patients scoring above a certain threshold on the anxiety scale would have an anxiety disorder and all patients scoring below that threshold would not. In practice, of course, the concordance is rarely 100%. In the present study there was evidence that very high anxiety scores not always implied a diagnosable anxiety disorder, and, conversely, that very low anxiety scores did not always imply the absence of anxiety disorder diagnosis. A possible reason for high anxiety scores in the absence of an anxiety disorder diagnosis might be that the patient did not fulfil all necessary criteria for a diagnosis (regarding e.g., duration, distress or disability). A possible reason for low anxiety scores in the presence of anxiety disorder might be that in some anxiety disorder cases manifest anxiety (as measured by the 4DSQ) was not a prominent feature of the disorder or was not necessarily present all the time. This happened relatively more often in cases diagnosed as GAD or specific phobia.

The observed association between the anxiety score and the diagnosis of anxiety disorder is also determined by the amount of measurement error, both in the anxiety score and in the assessment of the anxiety disorder diagnosis. Measurement error in the anxiety disorder diagnosis translates into misclassification and reduced reliability of the diagnosis. In our studies diagnostic reliability was not assessed, but typically the interrater agreement (Cohen’s kappa) of anxiety disorder diagnoses varies between 0.60 and 0.80 [41]. A kappa of 0.70 means 70% agreement after correction for chance agreement. Considering that there is a continuity between normality and anxiety disorder, it should be realized that the risk of misclassification is greatest near the threshold of disorder.

The mean reliability of the anxiety score across the study samples was 0.90, yielding a SEM of 2 points, suggesting that the 84% confidence interval of a given observed anxiety score X was X ± 3. In other words, when the observed anxiety score was X, we could be at least 92% confident that the true score was not > (X + 3) and we could also be at least 92% confident that the true score was not < (X–3).

When performing ROC analyses, we observed wide confidence intervals and significant variability of the thresholds across the studies. This variability must be attributed to differences in prevalence and severity spectrum of the samples, and also to distributional irregularities produced by chance in relatively small samples. Combining the samples by pooling was a logical action in order to obtain more stable estimates. This way we obtained 6.5 as the best single threshold to detect panic disorder, agoraphobia, social phobia, OCD and PTSD. Yet, using this single threshold would misclassify over a quarter of patients in either group. Therefore, we chose two thresholds, one (3.5) with a relatively high sensitivity to single out patients with a relatively low probability of having panic disorder, agoraphobia, social phobia, OCD or PTSD and one threshold (9.5) with a relatively high specificity to single out patients with a relatively high probability of having panic disorder, agoraphobia, social phobia, OCD or PTSD. Note that both thresholds are 1.5 SEM away from the threshold (6.5) of panic disorder, agoraphobia, social phobia, OCD and PTSD. This implies that we can be more than 92% confident that patients with anxiety scores 0–3 do not have a true anxiety score above the threshold of panic disorder, agoraphobia, social phobia, OCD and PTSD. Conversely, we can be more than 92% confident that patients with anxiety scores 10–24 do not have a true anxiety score below the threshold of panic disorder, agoraphobia, social phobia, OCD and PTSD. The uncertainty about whether or not a patient has passed the threshold of panic disorder, agoraphobia, social phobia, OCD and PTSD has now been restricted to one third (25-42%) of all patients, who score 4–9 on the anxiety scale.

The primary care professional can use the two cut-off points of the 4DSQ anxiety scale to separate patients with mental health problems into three groups: (1) a group with high anxiety scores (10–24), (2) a group with moderate scores (4–9), and (3) a group with low scores (0–3). Patients with high anxiety scores have a relatively high probability of having one or more anxiety disorders. Importantly, a high anxiety score does not represent a clinical diagnosis in itself. In addition, as noted earlier, the 4DSQ anxiety score does not indicate which specific anxiety disorder(s) is (are) present. A clinical diagnosis should be made in the short term using clinical judgment and available clinical guidelines [48,49]. Given the likelihood ratio, the chance of diagnosing one or more anxiety disorders is relatively high. Moreover, patients with high anxiety scores tend to have relatively clear-cut disorders as most borderline anxiety disorders are classified into the moderate group. On the other hand, patients with low anxiety scores have a low probability of anxiety disorder and when they do have an anxiety disorder, it will often be GAD or specific phobia, or a borderline anxiety disorder. These patients do not need a diagnostic interview targeted at anxiety disorder for the time being. Probably, in this low anxiety scores group, other problems (e.g., depression, stressful life situations) are more important to address. In the middle group with moderate anxiety scores (which constituted one third of our pooled sample), the possibility of anxiety disorder has not been ruled out as the probability is about the same as the prevalence. Anxiety disorder cases in this group are relatively often just beyond the diagnostic threshold and other problems (e.g., depression, stress) might be in more need of treatment. We argue that a diagnostic workup targeted at anxiety disorder can be postponed for a few weeks while monitoring the effect of non-specific interventions (e.g., reassurance, encouragement, advice) and the passage of time. When after 3–4 weeks symptoms decline, further diagnostic workup targeted at anxiety disorder does not seem to be necessary, but when symptoms do not abate a diagnostic interview is warranted after all. In our experience, this way GPs can efficiently target their diagnostic efforts to patients with a relatively high risk of having an anxiety disorder while keeping patients with moderate risk under surveillance. We acknowledge that there is currently no firm evidence to support this strategy, but it is our impression that it works fine in the primary care setting. More research is needed in this area.

The main limitation of the present study relates to the representativeness of the datasets included. Each of the datasets had been collected for other purposes than to evaluate the measurement properties of the 4DSQ. We would have preferred a large representative sample of primary care patients with mental health problems, each extensively assessed using a standardized psychiatric interview. However, this is costly and logistically challenging. Therefore, we employed convenience datasets collected in other studies. We assumed that the psychiatric diagnoses were principally invariant across the study samples as the samples could all be considered draws from the same large pool of primary care patients with mental health problems. Due to sampling differences, a fair degree of heterogeneity across the studies was evident, but this probably represented a strength of our study instead of a weakness. Furthermore, as the 4DSQ anxiety scale demonstrated high reliability and identical measurement properties across the studies, we assumed that the operating characteristics of the scale (i.e., sensitivity and specificity) were principally the same across the studies, only varying due to sampling. Therefore, we assumed that pooling (i.e., effectively conducting a patient level meta-analysis) was the best way to obtain valid estimates for the operating characteristics of the anxiety scale.

A second limitation concerns the fact that some studies did not assess the whole range of anxiety disorders. Notably, specific phobia, OCD and PTSD were not included in three studies. We estimate that if these diagnoses would have been established with a prevalence of 10-15%, assuming that at least two thirds of these disorders would co-occur with another (already known) anxiety disorder, the total increase in anxiety disorder patients across the studies would amount to 5-10%. This would have lead to a small decrease in the anxiety scores of patients without anxiety disorder. We assume that this would not have changed the results in any substantial way. However, replication in new samples would be desirable.

A third limitation constitutes the lack of information about interrater reliability of the diagnostic interviews. We relied on the reported reliability of these standardized interviews when performed by carefully trained interviewers. However, it should be noted that low reliability (i.e., measurement error) would attenuate existing associations between the 4DSQ anxiety score and anxiety disorder diagnosis. Because measurement error usually does not correlate with anything, it is unlikely that low reliability would be responsible for false associations. In other words, the associations in this study, as expressed in areas under the ROC-curve, sensitivities, specificities and likelihood ratios, are real and provide some reassurance regarding the diagnostic reliability.

This study took place in the DSM-IV era. However, in the meantime the DSM-5 – published in May 2013 – has decided not to classify OCD and PTSD as anxiety disorders anymore [50]. Instead OCD is included in a separate section with disorders characterized by compulsive behaviour, whereas PTSD is included in a section with disorders following traumatic or stressful events. Yet, our findings provide evidence of at least some degree of kinship between these disorders and typical anxiety disorders like panic disorder, agoraphobia and social phobia.

## Conclusions

The 4DSQ anxiety scale measures a common trait of pathological anxiety that is characteristic of the anxiety disorders, in particular panic disorder, agoraphobia, social phobia, OCD and PTSD. This property enables the anxiety scale to distinguish between patients with high risk of having an anxiety disorder (especially panic disorder, agoraphobia, social phobia, OCD and PTSD) and patients with low risk. It should be noted that the 4DSQ anxiety score is not able to distinguish between the separate anxiety disorder types. We propose to use ≥4 and ≥10 as cut-off points. Scores ≥4 should serve as a prompt to consider the possible presence of an anxiety disorder (while the probability is still relatively low), whereas scores ≥10 serve best as a prompt to pursue a clinical diagnostic workup for anxiety disorder immediately (as the probability is relatively high).

## Abbreviations

4DSQ: Four-dimensional symptom questionnaire; AUC: Area under the curve; BAI: Beck anxiety inventory; CFA: Confirmatory factor analysis; CFI: Comparative fit index; CIDI: Composite International Diagnostic Interview; DASS: Depression anxiety stress scale; DSM-IV: Diagnostic and Statistical Manual of Mental Disorders, 4^th^ edition; GAD: Generalized anxiety disorder; GAD-7: Generalized anxiety disorder scale; GP: General practitioner; HADS: Hospital Anxiety and Depression Scale; ICD-10: International Classification of Diseases, 10^th^ edition; MASQ: Mood and anxiety symptom questionnaire; npv: Negative predictive value; MINI: Mini-International Neuropsychiatric Interview; LR: Likelihood ratio; OCD: Obsessive-compulsive disorder; ppv: Positive predictive value; PTSD: Posttraumatic stress disorder; RMSEA: Root mean square error of approximation; ROC: Receiver operating characteristic; TLI: Tucker-Lewis Index.

## Competing interests

BT is the copyright owner of the 4DSQ and receives copyright fees from companies that use the 4DSQ on a commercial basis (the 4DSQ is freely available for non-commercial use in health care and research). BT received fees from various institutions for workshops on the application of the 4DSQ in primary care settings. The other authors declare that they have no competing interests.

## Authors’ contributions

BT and HvM conceived the idea for the manuscript. BT drafted the manuscript. PvdV performed the confirmatory factor analysis. BT performed the other statistical analyses. EB acquired the data of study A and assisted in the analyses of these data. AvS acquired the data of study C and assisted in the analyses of these data. DO acquired the data of study D and assisted in the analyses of these data. WL acquired the data of study E and assisted in the analyses of these data. All authors participated in the critical revision and interpretation of the data, and read and approved the final manuscript.

## Pre-publication history

The pre-publication history for this paper can be accessed here:

http://www.biomedcentral.com/1471-244X/14/121/prepub
